# The Metabolomic Characteristics and Dysregulation of Fatty Acid Esters of Hydroxy Fatty Acids in Breast Cancer

**DOI:** 10.3390/metabo13111108

**Published:** 2023-10-24

**Authors:** Linlin Qin, Na An, Bifeng Yuan, Quanfei Zhu, Yuqi Feng

**Affiliations:** 1 Department of Chemistry, Wuhan University, Wuhan 430072, China; 2018102030010@whu.edu.cn (L.Q.); 2017202030018@whu.edu.cn (N.A.); 2 School of Public Health, Wuhan University, Wuhan 430071, China; bfyuan@whu.edu.cn; 3 Frontier Science Center for Immunology and Metabolism, Wuhan University, Wuhan 430071, China

**Keywords:** FAHFA, breast cancer, chemical isotope labeling, liquid chromatography, mass spectrometry

## Abstract

Lipid reprogramming metabolism is crucial for supporting tumor growth in breast cancer and investigating potential tumor biomarkers. Fatty acid esters of hydroxy fatty acids (FAHFAs) are a class of endogenous lipid metabolites with anti-diabetic and anti-inflammatory properties that have been discovered in recent years. Our previous targeted analysis of sera from breast cancer patients revealed a significant down-regulation of several FAHFAs. In this study, we aimed to further explore the relationship between FAHFAs and breast cancer by employing chemical isotope labeling combined with liquid chromatography−mass spectrometry (CIL-LC-MS) for profiling of FAHFAs in tumors and adjacent normal tissues from breast cancer patients. Statistical analysis identified 13 altered isomers in breast cancer. These isomers showed the potential to distinguish breast cancer tissues with an area under the curve (AUC) value above 0.9 in a multivariate receiver operating curve model. Furthermore, the observation of up-regulated 9-oleic acid ester of hydroxy stearic acid (9-OAHSA) and down-regulated 9-hydroxystearic acid (9-HSA) in tumors suggests that breast cancer shares similarities with colorectal cancer, and their potential mechanism is to attenuate the effects of pro-apoptotic 9-HSA by enhancing the synthesis of FAHFAs, thereby promoting tumor survival and progression through this buffering system.

## 1. Introduction

Breast cancer (BC) is the most prevalent type of cancer among women and a leading cause of illness and death globally. In 2021, the World Health Organization estimated that there were 2.3 million new cases of BC, accounting for 12% of all newly diagnosed cancer cases. This number is projected to increase to 3.2 million annually by 2030. BC ranks as the fifth leading cause of death worldwide, with over 680,000 women dying from the disease in 2020, representing approximately 1 in 6 cancer deaths among women [1,2]. Despite significant progress in BC prevention and treatment, the increasing incidence rates indicate that it remains a substantial threat to women’s health. Therefore, extensive research is crucial to improve prevention, early diagnosis, and treatment strategies for BC.

Lipid reprogramming metabolism plays a crucial role in the occurrence and development of BC [3]. BC cells obtain energy for proliferation through abnormal lipid synthesis and degradation while accumulating metabolic products favorable for growth and survival [4]. One class of lipid molecules, fatty acid esters of hydroxy fatty acids (FAHFAs), is synthesized mainly in subcutaneous white adipose tissue. Specific FAHFAs have been shown to improve glucose tolerance, enhance insulin sensitivity, and exhibit anti-inflammatory effects, making them potential therapeutic targets for type 2 diabetes (T2D) [5,6]. T2D and obesity are risk factors that promote the development of BC, and obesity-related inflammation exacerbates BC progression [7,8,9]. Subcutaneous adipose tissue, a major component of the breast, serves as a lipid-rich microenvironment and supports the proliferation of BC cells by inflammation-induced lipolysis [10,11]. The disorder metabolism of FAHFAs was observed in the milk of obese mothers, suggesting de novo lipogenesis including the synthesis of FAHFAs was influenced by the abnormal status of adipocytes in the mammary gland [12]. Our previous study observed lower levels of FAHFAs in the serum of breast cancer patients compared to healthy individuals, suggesting alterations in FAHFA metabolism in BC patients [13]. It is speculated that FAHFAs possess anti-diabetic and anti-inflammatory properties that may be closely linked to the development of breast cancer.

Meanwhile, some progress has been made regarding the investigation of the role of FAHFAs in colorectal cancer (CRC), and it has been suggested that FAHFAs may have beneficial effects against cancer development [14]. FAHFAs may be biomarkers for the diagnosis of early CRC and show a reduced effect on the severity of colorectal ulcers, and they have the potential to decrease the risk of developing CRC [15,16]. Rodriguez et al. [17] showed elevated levels of 9-hydroxy stearic acid (9-HSA)-containing FAHFA in colorectal tumors compared to normal tissue. The addition of 9-HSA in in vitro cell lines resulted in increased FAHFA synthesis. In contrast to 9-HSA, which induces apoptotic effects in colorectal cancer cells, FAHFA demonstrated non-apoptotic effects [17]. Thus, the observations suggest that the synthesis of FAHFA can be a buffering system that limits apoptosis by sequestering hydroxy fatty acid (HFA) into an inactive form [17]. Both CRC and BC are malignant tumors with a hallmark feature of lipid reprogramming [18]. However, we still have little knowledge of the dysregulation of FAHFAs in BC in comparison to CRC.

In this study, we screened and quantitatively analyzed FAHFAs in 24 pairs of BC tissues and adjacent non-cancerous tissues to investigate the alterations of FAHFA metabolism in BC. FAHFAs were detected using chemical isotope labeling combined with liquid chromatography−mass spectrometry (CIL-LC-MS) strategy. This strategy employed *N*, *N*-dimethylethylenediamine (DMED/*d_4_*-DMED), a pair of carboxyl isotope labeling reagents, to enhance the sensitivity and specificity of FAHFA detection. Subsequently, multivariate statistical analysis and receiver operating characteristic curves (ROC) were applied to identify significantly changed FAHFAs in BC and evaluate the predictive model performance for distinguishing BC. Our study contributes to a better understanding of lipid metabolism in BC.

## 2. Materials and Methods

### 2.1. Collection of Breast Tissue

Tissue samples from BC patients and their adjacent normal tissues were collected from Hubei Cancer Hospital in Wuhan, China. A total of 24 patients participated in the study, and their tissue samples were quickly stored in liquid nitrogen and kept at −80 °C for preservation. The tissue samples were obtained from female donors who had been diagnosed with breast cancer of the tumor type. The average age of these patients was 50 years, with the majority falling within the range of 40–60 years. Detailed information of 24 patients have been provided in Table S1, including the female donor’s age, gender, and diagnosis type of the cancer. The study was conducted following the Ethics Committee of Wuhan University’s guidelines and regulations.

### 2.2. Chemicals and Reagents

All FAHFA standards were purchased from Cayman Chemical (Ann Arbor, MI, USA), RC ChemTec Co., Ltd. (Wuhan, China), and XuKang Medical Science and Technology Co., Ltd. (Xiangtan, China). The full names of the FAHFAs can be found in Table S2 in the Supporting Material. The detailed information on FAHFA standards is listed in Table S3 in the Supporting Material. Analytical grade formic acid, ammonia, triethylamine (TEA), 2-chloro-1-methylpyridinium iodide (CMPI), and 2-dimethylamino ethylamine (DMED) were supplied by Sinopharm Chemical Reagent Co (Shanghai, China). *d_4_*-DMED was synthesized according to the method reported in our previous work [19]. HPLC-grade acetonitrile (ACN), isopropanol (IPA), acetone, chloroform, and methanol were purchased from Merck (Darmstadt, Germany). A strong anion exchange solid phase extraction column (SAX SPE column, 3 mL, 200 mg) was provided by Weltech Co. (Wuhan, China). Before use, water was purified with a Milli-Q device (Millipore, Bedford, MA, USA). CMPI, TEA, DMED, and *d_4_*-DMED were prepared in ACN and stored at −20 °C.

### 2.3. Sample Pretreatment

Total lipid extraction from BC tissues was performed using the method described by Folch with minor modifications [20,21]. Tissues were weighed and homogenized in 1 mL of methanol with a homogenizer for 1 min (×2 cycles). Next, chloroform in the amount of 2 mL was added and vortexed for 5 min. Then, 1 mL of injectable saline with 0.9% NaCl was added and vortexed for 30 s. The lipid extract was centrifuged at 15,000 rpm for 10 min at 4 °C. After centrifugation, the chloroform layer was transferred to a new Eppendorf centrifuge tube, and the aqueous layer was repeated with another 2 mL of chloroform. The combined chloroform layers containing lipids were collected and blow-dried under nitrogen. The dried and concentrated lipid extracts were re-dissolved with acetonitrile containing 0.1% ammonia, followed by removal of impurities using a SAX SPE column, as in our previous work [21]. SPE eluate was blow-dried under nitrogen, re-dissolved with acetonitrile, and divided into two equal portions. After the reaction, the solution was dried under nitrogen and stored at −20 °C before use.

### 2.4. Mass Analysis

The experiment used LC-ESI-MS/MS system, including the Shimadzu LC-20AD HPLC system (Tokyo, Japan) and Shimadzu MS-8050 mass spectrometer (Tokyo, Japan), equipped with an ESI ion source (Turbo Ion spray). The LC system had two 30AD pumps, a SIL-30AC autosampler, a CTO-20A thermostat column compartment, and a DGU-20A5R degasser. The chromatographic column for liquid phase separation was Acquity UPLC BEH C18 column (2.1 × 50 mm, 1.7 μm, Waters), the flow rate was 0.4 mL/min, the column temperature was 30 °C, and the injection volume was 10 μL. The mobile phases were (A) ACN/H_2_O (6/4, *v*/*v*) containing 0.1% formic acid and (B) IPA/ACN (9/1, *v*/*v*) containing 0.1% formic acid. The gradient used was as follows: 0-2 min 35% B, 2–30 min, 35–90% B, 30–33 min, 90% B, 33–34 min, 90–35% B, and 34–38 min, 35% B. The LC-MS screening and quantitative analysis were performed using multiple reaction modes (MRM) in positive ion mode. The method utilizes the cleavage pattern of DMED/*d_4_*-DMED-labeled FAHFA with a neutral loss (NL) of 63/67 Da and loss of fatty acid (FA) at specific collision energy to detect ^DMED^FAHFA/*^d4^*^-DMED^FAHFA. The loss of 63/67 Da was attributed to the elimination of N-dimethyl (NL 45 Da) and a molecule of H_2_O in [^DMED^FAHFA-FA]^+^. According to this pattern, [^DMED^FAHFA]^+^/[*^d4^*^-DMED^FAHFA]^+^ was the parent ion, whereas [^DMED^FAHFA-FA-63]^+/^[*^d4^*^-DMED^FAHFA-FA-67]^+^ was the daughter ion. In addition, the quantitative analysis added auxiliary ^DMED^FAHFA qualitative transition channels based on the secondary strongest daughter ion produced during ^DMED^FAHFA cleavage which was [^DMED^FAHFA-FA-45]^+^, i.e., [^DMED^FAHFA]^+^ > [^DMED^FAHFA-FA-45]^+^. The MRM mass spectrometer parameters of potential FAHFAs are listed in Table S4, and product ions spectra of DMED/*d_4_*-DMED-labeled FAHFAs is shown in Figure S1.

### 2.5. Statistical Analysis

The relative abundance of FAHFAs was determined by calculating the peak height ratio of ^DMED^FAHFA to that of the *^d4^*^-DMED^FAHFA standards. The relative abundance data of FAHFAs and HFAs in BC tissues were log10 transformed, z-score normalized, and subjected to multivariate and biomarker analysis. Retention index (RI) was used to calibrate retention time according to our previous method [22]. Orthogonal partial least squares discrimination analysis (OPLS-DA) was performed using SIMCA 14.1 software (Umetrics AB, Umea, Sweden). The significance of the differences was assessed using a nonparametric univariate Student’s *t*-test, with a *p*-value threshold of less than 0.05. Additionally, the *p*-values were adjusted for multiple comparisons using the classical one-step method of false discovery rate (FDR). The volcano plot and Venn diagram were analyzed and plotted using OriginPro 2021 (OriginLab Corporation, Northampton, MA, USA). Multivariate receiver operating characteristic (ROC) tests were performed in MetaboAnalyst 5.0 (https://www.metaboanalyst.ca access date 3 May 2023). Violin plots were generated using GraphPad Prism 9.2.0 (GraphPad Software, San Diego, CA, USA).

## 3. Results

### 3.1. Screening and Annotation of FAHFAs in BC Tissue 

**Figure 1:**
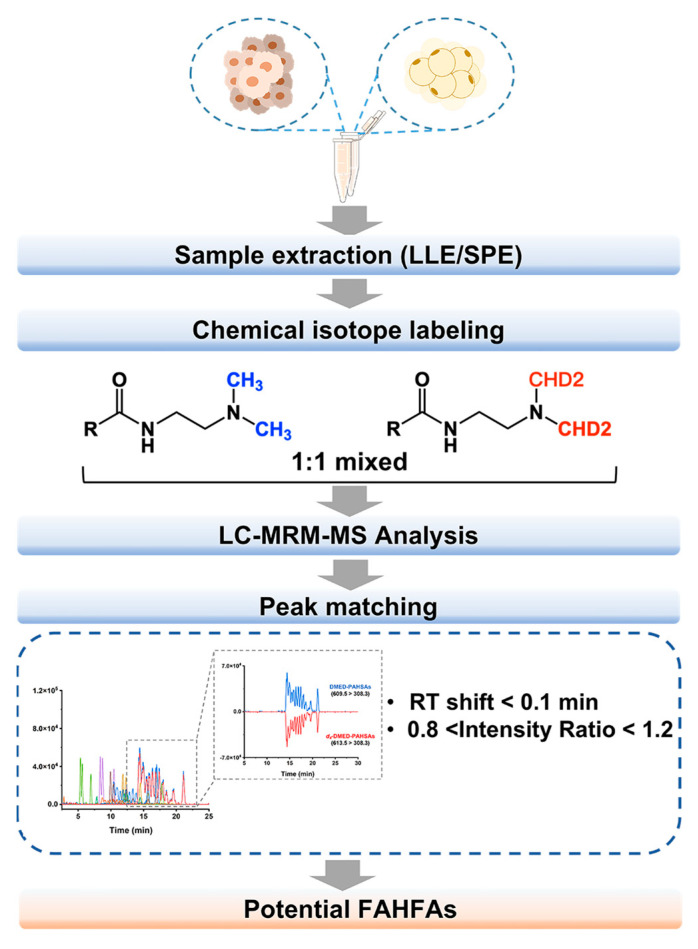
Schematic flow diagram of CIL-LC-MS screening of FAHFAs in breast tumor and adjacent normal tissues.

In this study, we employed CIL-LC-MS strategy to screen FAHFAs in BC tumors and adjacent normal tissues. The experimental workflow is outlined in Figure 1. Lipids were extracted from both tissue types obtained from different patients and divided into two aliquots for each type. The aliquots were labeled with DMED and *d_4_*-DMED, respectively, and then, equal volumes of the labeled samples were mixed for LC-MS analysis. FAHFAs were screened in MRM mode, and MRM transitions were created based on the fragmentation patterns of labeled FAHFAs. DMED/*d_4_*-DMED-labeled FAHFAs exhibit similar molecular structures and undergo ester bond cleavage and characteristic NL 63/67 Da under specific collision energy. As a result, pairs of FAHFAs with a mass difference of 4 Da showed identical retention time and signal intensity of chromatographic peaks. The chromatographic peak pairing of DMED/*d_4_*-DMED-FAHFAs was achieved by extracting ion chromatograms, and only peak pairs fulfilling the criteria mentioned above were considered potential FAHFA isomers. By employing this approach, we detected a total of 9 FAHFA families, including 72 isomers in breast tissues (Table S5).

**Figure 2:**
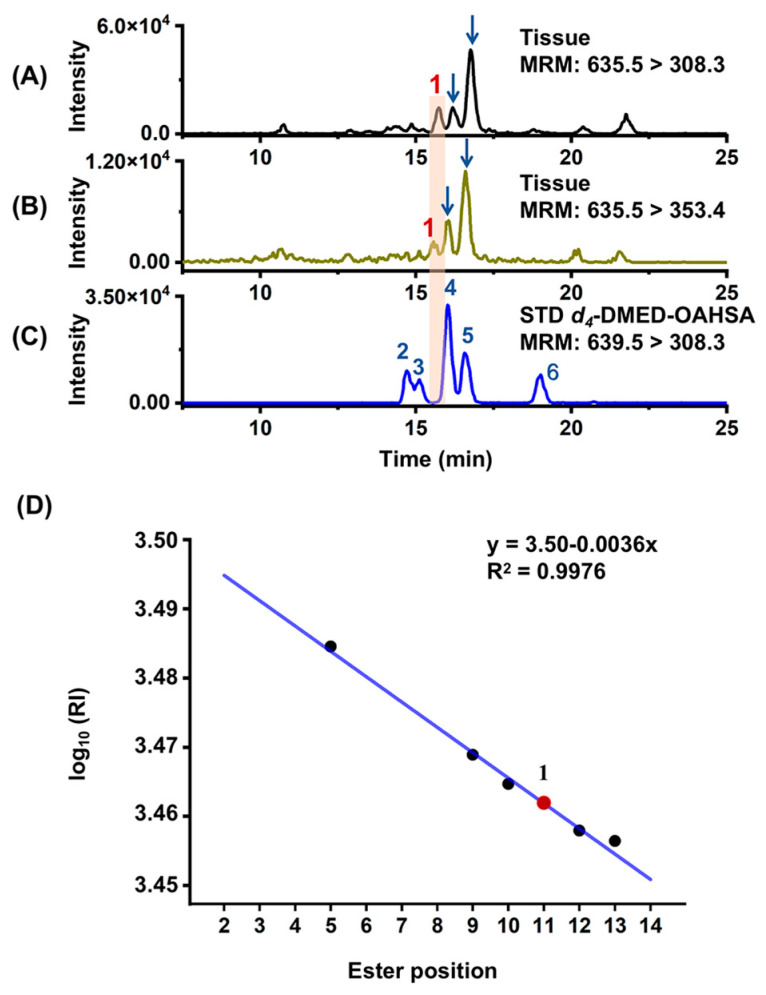
Identification of OAHSA isomers using the retention index ester bond position rule. The upper panel shows the extracted ion chromatograms of OAHSA in (**A**) tumor tissues, (**B**) adjacent normal tissues, and (**C**) standards (Peak 2, 13−OAHSA; Peak 3, 12−OAHSA; Peak 4, 10−OAHSA; Peak 5, 9−OAHSA; and Peak 6, 5−OAHSA), respectively. Regression curves of log_10_(RI) (RI, retention index) versus the ester bond positions of OAHSA (**D**) were constructed with OAHSA standards (dark blue dots), and **1** (red dot) represents the predicted 11-OAHSA.

The detected FAHFAs were identified based on standard confirmation or were putatively annotated based on chromatographic retention rules. Firstly, the MRM method was employed to screen for potential FAHFA families and obtain information about the FA and HFA components that constitute a particular FAHFA, facilitating family assignments. Subsequently, the molecular structures of specific isomers within the same family were determined using authentic standards, while unknown isomers for which no available standards were annotated based on their chromatographic RI relative to the ester bond position. For example, as shown in Figure 2, in the tumor tissue, three compounds were detected in two MRM channels (transition of 635.5 > 308.5 and 635.5 > 353.5) of oleic acid ester of hydroxy stearic acid (OAHSA). This indicates that these three compounds can be classified as OAHSAs. Among them, the retention times of the two compounds aligned with the blue arrows matched those of standards 4 and 5, confirming their structures as 10-OAHSA and 9-OAHSA, respectively. However, isomer **1** exhibited a retention time between the peak of standard 12-OAHSA (peak 3) and 10-OAHSA (peak 4), and no corresponding standard was available to confirm its structure. To determine the ester bond position of isomer **1**, a linear regression curve (y = 3.50 − 0.0036x, R^2^ = 0.9976) was constructed between the log_10_RI and ester bond position based on existing OAHSA standards (13-OAHSA, 12-OAHSA, 10-OAHSA, 9-OAHSA, and 5-OAHSA). Using the curve equation, the ester bond position of isomer **1** was predicted to be at the 11 position, thus identifying it as 11-OAHSA. Similar methods were employed to determine the structures of the other detected isomers. 

### 3.2. FAHFA Alterations in Breast Cancer Tissue

The quantitative analysis of the detected FAHFAs in tissues was performed using the internal standard method. Specifically, *d_4_*-DMED-labeled FAHFA standards were spiked in DMED-labeled lipid extracts as internal standards. A semi-quantitative analysis was conducted using standards with similar ester bond positions from the same family as internal standards for isomers without authentic standards. The results of the quantitative analysis are presented in Table S7.

**Figure 3:**
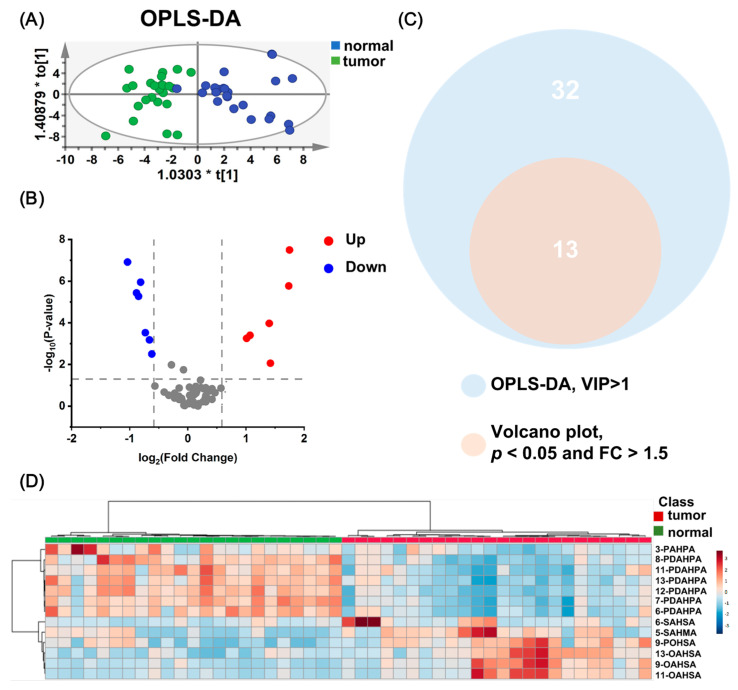
Statistical analysis of screening FAHFA isomers for breast cancer characteristics. (**A**) OPLS−DA score plots of FAHFAs show a clear separation between tumor tissue and normal tissue adjacent to tumor tissues. (**B**) Volcano plots display that 6 isomers are up−regulated and 7 isomers are down−regulated in tumor tissues, and fold change was calculated by the ratio of relative abundance of FAHFAs in tumor versus adjacent normal tissues. (**C**) Venn diagram shows that FAHFAs characteristic of breast cancer were obtained by screening for thresholds that were met both in OPLS−DA analysis (VIP > 1) and volcano plot analysis (*p* < 0.05, FC > 1.5). (**D**) Hierarchical clustering heatmaps show the relative abundance of 13 significantly differentiated FAHFAs in breast tissues, visualizing characteristic alteration of FAHFAs in tumor tissues and contributing to a precise classification of tissues.

Multivariate analysis techniques such as OPLS-DA and volcano plot analysis were employed to analyze the data further. The OPLS-DA score plot (Figure 3A) displayed a distinct separation between tumor and adjacent tissue, explaining 34.3% of the total model variance through the first two principal components. To ensure the reliability of the model, the R^2^ (less than 0.5) and Q^2^ (less than 0) values were evaluated, along with a permutation test (*n* = 200). The results (Figure S2A) showed that all permutation test values for R^2^ and Q^2^ were lower than the initial values, indicating that the model was not overfitted. Based on the variable importance in projection (VIP) values greater than 1 in the OPLS-DA model, 32 FAHFA isomers were selected (Figure S2B). Univariate analysis using volcano plots combined with Student’s *t*-test and fold change (FC) was used to analyze further metabolites that changed significantly in the sample. The *p*-values obtained from the student’s *t*-test underwent a false discovery rate (FDR) adjustment. Figure 3B illustrates 13 FAHFAs that exhibited fold changes (FC) greater than 1.5 and statistical significance (*p* < 0.05) between the two tissue types.

By intersecting the selection criteria from the volcano plot analysis and VIP values greater than 1, a Venn diagram analysis was constructed (Figure 3C), identifying 13 overlapping FAHFAs in the Venn diagram. These FAHFA isomers were designated as differential FAHFAs in BC. The VIP values, FC, and *p*-values for these metabolites are presented in Table 1. Hierarchical clustering heatmaps were generated to visualize the levels of FAHFAs in the two tissue groups. The proximity of the heatmap clusters indicates more minor differences in FAHFA levels between samples. Figure 3D shows the relative abundance of FAHFAs between the tumor and adjacent normal tissues for the FAHFA group screened using OPLS-DA and volcano analysis, which exhibited a good classification due to the isomers between tumor and normal tissues.

Interestingly, the violin plots (Figure S3) revealed that the up-regulated isomers in tumor tissue primarily consisted of mono-unsaturated OAHSAs and palmitoleic acid esters of hydroxy stearic acid (POHSAs). In contrast, the down-regulated isomers were predominantly saturated structured pentadecanoic acid esters of hydroxy palmitic acid (PDAHPAs) and palmitic acid esters of hydroxy palmitic acid (PAHPAs). These observed structural changes in FAHFAs associated with BC are consistent with previous findings in serum, which reported a significant down-regulation of seven isomers belonging to the saturated structural family PAHSA and stearic acid esters of hydroxy stearic acid (SAHSA) in the serum of BC patients. This further strengthens the validity of our findings [23].

**Figure 4:**
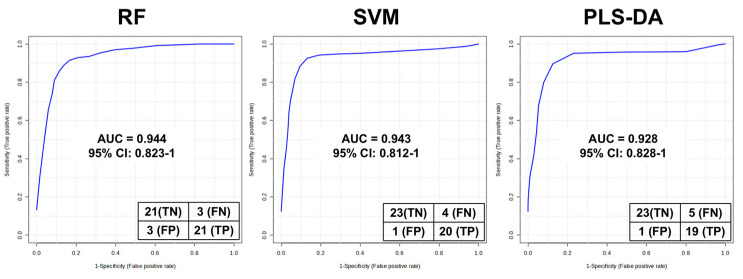
The multivariate ROC models were constructed using 13 differentiated FAHFAs, using three different machine learning methods, Random Forest (RF), Support Vector Machine (SVM), and Partial Least Squares Discriminant Analysis (PLS-DA), respectively.

The 13 BC-associated FAHFA isomer groups obtained from the statistical analyses underwent machine learning methods, including the Support Vector Machine (SVM), Random Forest (RF), and Partial Least Squares Discriminant Analysis (PLS-DA), to facilitate the classification of tumor and adjacent normal tissues. The Area Under the Curve (AUC) of ROC was used to evaluate the predictive performance of binary classification models. As depicted in Figure 4, all three methods yielded AUC values above 0.9, especially, an AUC of 0.944 for the RF model, suggesting the significant correlation between FAHFA and breast cancer. By selecting RF as the optimal method, several multivariate ROCs based on differentiated FAHFAs were constructed (Figure S4), and a three-variate model (AUC = 0.919) showed that 9-OAHSA, 11-OAHSA, and 12-PDAHPA could be characteristic isomers of FAHFAs’ dysregulation in BC (Figure S5).

### 3.3. Relationship between BC-Associated FAHFAs and HFAs

**Figure 5:**
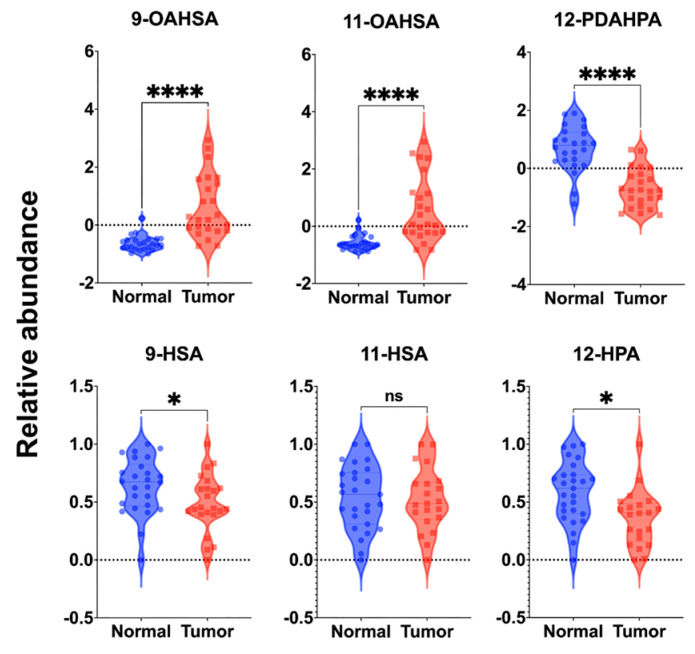
Violin scatter plots of characteristic FAHFAs and their related HFAs that can be used to diagnose breast cancer. *, *p* < 0.05; ****, *p* < 0.0001; ns, not significant.

Considering the significant role of HFAs as precursor molecules in FAHFAs biosynthesis, this study investigated the association between BC-associated FAHFAs and related HFAs. The relative abundance of HFAs in breast tissues was determined according to a previously reported LC-MS method [24] which is described in detail in the supporting information. 9-HSA, 11-HSA, and 12-hydroxy palmitic acid (12-HPA) were precursor HFAs of 9-OAHSA, 11-OAHSA, and 12-PDAHPA, respectively. As shown in Figure 5, while 11-HSA exhibited minimal content changes in tumor tissue, 9-HSA and 12-HPA showed substantial down-regulation. It is noteworthy that 9-HSA has been reported to promote apoptosis in colorectal cancer cells and is down-regulated in colorectal cancer, whereas the content of 9-OAHSA is significantly up-regulated. A similar trend observed in BC suggests that the sequestration of 9-HSA to 9-OAHSA could also be up-regulated in breast tumors. Furthermore, 11-OAHSA demonstrated a significant up-regulation in BC, while changes in 11-HSA were less pronounced. Alternatively, in comparison to mono-unsaturated OAHSA isomers, PDAHPA isomers generally exhibited down-regulation in tumor tissue, with 12-HPA also demonstrating a decreasing trend.

## 4. Discussion

In breast cancer (BC), cancer cells manipulate the secretion pattern of nearby adipocytes by releasing pro-inflammatory substances. This leads to increased breakdown of fats, providing the necessary energy for cancer cell growth and movement. Previous studies have explored the potential of anti-inflammatory drugs and identified inflammatory metabolic pathways as possible targets for therapy [25,26]. Research has also shown that specific FAHFA metabolites, including 9-PAHSA, 13-DHAHLA, and 13-LAHLA, can inhibit the expression of inflammatory cytokines [27,28,29]. Studies focusing on serum FAHFAs in BC patients have found significantly reduced levels of the four isomers, including 9-PAHSA [23]. Based on these findings, this study aimed to investigate changes in FAHFAs in both tumor tissue and adjacent normal tissue in BC patients.

The study identified a total of nine families, including 72 isomers, and observed significant changes in the abundance of 13 isomers. Among these, seven isomers from the families of OAHSAs, POHSAs, SAHMAs, and SAHSAs were up-regulated, while six isomers from the families of PDAHPAs and PAHPAs were down-regulated. The evaluated ROC models for specificity and sensitivity of potential biomarkers showed that the models based on 9-OAHSA, 11-OAHSA, and 12-PDAHPA had AUC values greater than 0.9, indicating a strong correlation between FAHFA and BC.

In BC tissue, elevated levels of OAHSAs, associated with monounsaturated fatty acids, were observed compared to normal tissue. Conversely, PDAHPAs, associated with saturated fatty acids, displayed the opposite trend. Specifically, the characteristic biomarkers of BC, 9-OAHSA, and 11-OAHSA showed significant up-regulation, while 12-PDAHPA was down-regulated. A previous study has reported that the serum levels of seven FAHFAs (13-PAHSA, 12-PAHSA, 9-PAHSA, 5-PAHSA,13-SAHSA, 12-SAHSA, and 9-SAHSA) in BC patients were found to be lower compared to those in healthy individuals. Slight differences were observed between breast cancer tissues and serum. The above seven isomers were decreased in serum and did not show significant changes in tissues. Additionally, the OAHSA and POHSA families did not exhibit significant alterations in serum, but several isomers (9-OAHSA, 11-OAHSA, 13-OAHSA, and 9-POHSA) showed an increase in breast cancer tissues. In serum, 16 FAHFAs (PAHSAs, POHSAs, SAHSAs, and OAHSAs) were targeted for quantification, and the observed significantly altered isomers of 7 FAHFAs were all down-regulated. Nevertheless, the scope of families detected in tissue increased, and those found to be significantly changed in breast cancer were both up- and down-regulated. These differences in serum and tissue findings may be due to the fact that serum reflects overall levels of circulating metabolites, whereas tissue analyses highlight changes between lesions and adjacent tissues. Although there were differences in the families and isomers that appeared altered in serum and tissue, the commonality of observations suggests that saturated structural FAHFAs are down-regulated in breast cancer patients. 

It is worth noting that the increased presence of 9-OAHSA may be associated with tumor-induced inflammation. Recent studies have shown that 9-OAHSA can reduce the expression of tumor necrosis factor-α (TNF-α), interleukin 6 (IL6), chemokine ligand 2 (CCL2), CCL3, and CCL5 induced by lipopolysaccharide (LPS) and enhance macrophage phagocytosis [30]. This suggests that 9-OAHSA may be linked to the modulation of inflammatory responses in BC. However, further research is needed to explore the biological activities of 11-OAHSA and 12-PDAHPA and their roles in BC.

In the currently proposed mechanisms for FAHFAs synthesis, HFAs play a crucial role as precursor molecules [31,32]. Consequently, we measured the levels of HFAs associated with three potential diagnostic markers for BC. The levels of 9-HSA were found to be lower in BC tissue compared to adjacent normal tissue, consistent with previous observations for colorectal cancer tissue [17]. Based on these findings, our hypothesis suggests that BC cells may employ a strategy similar to those of colorectal cancer cells, converting the pro-apoptotic molecule 9-HSA into neutral FAHFAs. This conversion establishes a buffering system that slows down cell apoptosis. Therefore, it is plausible that BC cells utilize such a buffering system to limit the pro-apoptotic effects of 9-HSA.

The variations in 11-HSA levels in tumors were insignificant, while the up-regulation of 11-OAHSA may primarily be attributed to increased oleic acid (OA) levels. Previous studies have demonstrated the significant activation of stearoyl-CoA desaturase (SCD) in BC brain metastasis. This activation leads to the conversion of palmitic acid (PA) into OA through elongation and desaturation reactions. BC cells utilize this OA for proliferation and migration [33,34]. However, whether free fatty acids are involved in FAHFA synthesis remains unclear. Some studies have suggested that the fatty acid sources for FAHFA synthesis can originate from triglycerides (TAG) or diglycerides (DAG) [32]. Under the catalysis of adipose triglyceride lipase (ATGL), these fatty acids undergo acylation with HFA to synthesize FAHFA. Other studies have indicated that dietary intake of fatty acids may influence the synthesis of plasma FAHFA. For example, Kuda et al. found that individuals supplemented with omega-3 polyunsaturated fatty acids (docosahexaenoic acid or eicosapentaenoic acid) exhibited significantly increased levels of docosahexaenoic acid esters of hydroxy linoleic acid (DHAHLA) compared to non-supplemented individuals [35]. Kelerer et al. observed a higher diversity of FAHFA in the plasma of omnivores compared to vegans, and young males showed a significant increase in saturated FAHFA levels after the intake of saturated fat (1000 kcal/week) [36]. Nonetheless, further investigation is required to accurately determine whether there are alterations in the synthesis pathways of specific FAHFAs in BC. Additional research is necessary to fully elucidate the mechanisms underlying FAHFA synthesis in BC cells.

In our study, we observed a significant down-regulation of 12-HPA in tumors, while 12-PDAHPA did not exhibit a similar up-regulation trend as seen with 9-OAHSA. Instead, its content was significantly decreased in tumor tissues. This suggests that 12-HPA may be consumed during the synthesis process of 12-PDAHPA and is likely rapidly converted into other metabolites. Previous research indicates that FAHFAs may exist in the form of FAHFA-TAG stored in TAG, and they are only released during fasting or starvation [37]. Therefore, the synthesized 12-PDAHPA may be eventually converted into other forms, such as FAHFA-TAG. To verify this hypothesis, further research is needed to delineate the metabolic pathways of 12-PDAHPA and its specific fate in tumors. These studies will provide initial information and insights into the role of 12-PDAHPA in tumor development.

Several limitations should be acknowledged for this study. Firstly, the sample size was limited, with only 24 pairs of tissue samples from BC patients being analyzed. This may limit the generalizability of the study findings. Secondly, detailed staging of the patients was not conducted, which could impact the interpretability of metabolic differences between different stages of BC. Additionally, this study focused only on specific metabolites and did not comprehensively analyze the entire metabolic network of BC. Therefore, further research with larger sample sizes, consideration of staging information, and comprehensive analysis of multiple metabolic pathways is necessary to gain a comprehensive understanding of the relevance of BC metabolism.

## 5. Conclusions

In this study, a CIL-LC-MS strategy was employed to analyze FAHFAs in BC tumors and adjacent normal tissues. The study revealed that 13 FAHFAs were significantly altered in BC patients, with 3 FAHFAs showing a potential for being possible biomarkers for BC. Similar to colorectal cancer, studies in BC tissues suggest that tumor cells may attenuate apoptosis by converting 9-HSA into 9-OAHSA. However, further research is needed to investigate the biological activities of these lipid metabolites and their roles in BC. Despite certain limitations in this study, it has provided a fundamental basis for understanding alterations in lipid metabolism in BC, early diagnosis, and treatment. Future studies could provide a more comprehensive understanding of the relevance of breast cancer metabolism by expanding the sample size and comprehensively analyzing more metabolic pathways.

## Figures and Tables

**Table 1:** Differentiating FAHFAs between breast cancer tumors and adjacent normal tissues.

No.	Analytes	VIP ^1^	FC ^2^	log_2_(FC)	*p*-Value ^3^	FDR ^4^	log_10_ (*p*-Value)	Regulation
1	9-OAHSA	1.5	3.2	1.7	3.2 × 10^−8^	2.0 × 10^−5^	7.5	up
2	11-OAHSA	1.5	3.2	1.7	1.7 × 10^−6^	1.8 × 10^−4^	5.8	up
3	5-SAHMA	1.3	2.6	1.4	1.1 × 10^−4^	1.4 × 10^−3^	4.0	up
4	13-OAHSA	1.3	2.1	1.1	1.2 × 10^−4^	8.3 × 10^−4^	3.9	up
5	9-POHSA	1.1	2.0	1	5.6 × 10^−4^	1.7 × 10^−3^	3.3	up
6	6-SAHSA	1.3	2.6	1.4	8.8 × 10^−3^	1.6 × 10^−2^	2.1	up
7	12-PDAHPA	1.6	0.5	−1	1.2 × 10^−7^	2.6 × 10^−6^	6.9	down
8	7-PDAHPA	1.5	0.6	−0.8	1.1 × 10^−6^	2.1 × 10^−5^	5.9	down
9	8-PDAHPA	1.6	0.5	−0.9	3.7 × 10^−6^	2.7 × 10^−5^	5.4	down
10	13-PDAHPA	1.4	0.6	−0.8	5.4 × 10^−6^	2.0 × 10^−5^	5.3	down
11	6-PDAHPA	1.3	0.6	−0.7	3.0 × 10^−4^	8.3 × 10^−4^	3.5	down
12	11-PDAHPA	1.4	0.6	−0.7	6.6 × 10^−4^	2.7 × 10^−3^	3.2	down
13	3-PAHPA	1.0	0.7	−0.6	3.2 × 10^−3^	1.7 × 10^−2^	2.5	down

^1^ Variable importance in projection (VIP) is obtained from the OPLS-DA with a threshold of 1.0. ^2^ Fold change (FC) was calculated by the average value of breast cancer tumor tissue versus adjacent normal tissues. FC values greater than zero indicate higher levels of FAHFAs in tumor tissue compared to adjacent normal tissues, while FC values less than zero indicate lower levels. ^3^ The *p*-value was calculated with Student’s *t*-test. ^4^ False Discovery Rate (FDR)-adjusted *p*-value.

## Data Availability

All data that support the findings of the study are within the manuscript or in the Supplemental Materials.

## Supplementary Materials

The following supporting information can be downloaded at: https://www.mdpi.com/article/10.3390/metabo13111108/s1, Table S1: Characteristics of study participants; Table S2: The abbreviations of lipid compounds; Table S3: List of FAHFA standards; Table S4: The MRM parameters for FAHFA candidates with LC-ESI-MS/MS; Table S5: Detected FAHFAs in tissues; Table S6: Structures of FAHFAs detected in breast tissues; Table S7: Measured levels of FAHFAs detected in breast tissues, including absolute quantitative and semi-quantitative analysis; Figure S1: Product ions spectra of DMED/*d_4_*-DMED-labeled FAHFAs; Figure S2: Permutation and VIP distribution of OPLS-DA module; Figure S3: The violin scatter plots of FAHFA isomers with significant differences in tumor tissue and adjacent normal tissue in cancer; Figure S4: Multivariate ROC model based on differentiated FAHFAs using Random Forest as a construction method; Figure S5: Multivariate ROC analysis based on 9-OAHSA, 11-OAHSA, and 12-PDAHPA.
