# Supplementary material for: The Metabolomic Characteristics and Dysregulation of Fatty Acid Esters of Hydroxy Fatty Acids in Breast Cancer
Source: Metabolites. 2023 Oct 24;13(11):1108. doi: 10.3390/metabo13111108 (PMC10673550; doi:10.3390/metabo13111108)
Supplement: Supplementary file 1 [file metabolites-13-01108-s001.zip › metabolites-2666484-supplementary.pdf]

Supporting information

The Metabolomic Dysregulation of Fatty Acid Esters of Hydroxy Fatty Acids in Breast Cancer

Linlin Qin [1], Na An [1], Bifeng Yuan [2], Quanfei Zhu [2,*] and Yuqi Feng [1,2,3,*]

[1] Department of Chemistry, Wuhan University, Wuhan 430072, China; 2018102030010@whu.edu.cn (L.Q.); 2017202030018@whu.edu.cn (N.A.)
[2] School of Public Health, Wuhan University, Wuhan 430071, China; bfyuan@whu.edu.cn
[3] Frontier Science Center for Immunology and Metabolism, Wuhan University, Wuhan 430071, China
* Correspondence: yqfeng@whu.edu.cn (Y.F.); qf_zhu@whu.edu.cn (Q.Z.)

The Supporting Information includes the following items:

Page S3-S4	Relative quantitation of hydroxy fatty acids
Page S5	Table S1. Characteristics of study participants
Page S6	Table S2. The abbreviations of lipid compounds
Page S7-S8	Table S3. List of FAHFA standards
Page S9-S10	Table S4. The MRM parameters for FAHFA candidates by LC-ESI-MS/MS.
Page S11-S14	Table S5. Detected FAHFAs in tissues.
Page S15-S16	Table S6. Structures of FAHFAs detected in breast tissues
Page S17-S18	Table S7.Measured levels of FAHFAs were detected in breast tissues, including absolute quantitative and semi-quantitative analysis.
Page S19	Figure S1. Product ions spectra of DMED/d_4-DMED labeled FAHFAs.
Page S20	Figure S2. Permutation and VIP distribution of OPLS-DA module.
Page S21	Figure S3. The violin scatter plots of FAHFAs isomers with significant differences in tumor and adjacent normal tissue of cancer.
Page S22	Figure S4. Multivariate ROC model based on differentiated FAHFAs using Random Forest as a construction method.
Page S23	Figure S5. Multivariate ROC analysis based on 9-OAHSA, 11-OAHSA, and 12-PDAHPA

Relative quantitation of hydroxy fatty acids

Chemical isotope labeling combined with liquid chromatography-mass spectrometry (CIL-LC-MS) was used to quantify HFAs in tissues. The experiments were performed on an AB Sciex 4500 triple quadrupole mass spectrometer (Massachusetts, USA) and a Shimadzu LC-20A system (Shimadzu, Japan). The system is equipped with LC-20AD dual pumps, SIL-20A autosampler, CTO-20AC constant temperature column oven, and DGU-20A3 in-line degasser. An Acquity UPLC BEH C18 column (2.1×50 mm, 1.7 μm, Waters) was used. The temperature of the column chamber was set at 40℃. The formic acid aqueous solution was used as mobile phase A (0.1%, v/v) and acetonitrile as mobile phase B at a 0.4 mL/min flow rate. The chromatographic gradients were: 0-3 min 10% B, 3-15 min 10 - 90% B, 15-25 min 90% B, 25-27 min 90 - 10% B, and 27-30 min 10% B. All labeled products were analyzed in positive ion mode. MRM analysis was performed using positive ion mode. Optimal ion source conditions: curtain gas 35 L/min, collision gas 8 L/min, ionization voltage 5500 V, ion source temperature 500 °C, spray gas 150 L/min, auxiliary heating gas 250 L/min.

The transitions of $[^{DMED}HFA]^+ \rightarrow [^{DMED}HFA\text{-}63]^+$ and $[^{d4\text{-}DMED}HFA]^+ \rightarrow [^{d4\text{-}DMED}HFA\text{-}67]^+$ for DMED and d_4-DMED-labeled HFA, respectively, were used as MRM ion pairs. d_4-DMED-labeled HFA standards were added as internal standards to assist the qualitative and quantitative analysis. Based on the chromatographic retention pattern of the hydroxyl position of HFA and the MRM channel with the characteristic diagnostic ions as product ions, the retention times of 9-HSA, 11-HSA, and 12-HPA were determined and quantitatively

analyzed. The relative quantification of HFA was obtained by calculating the peak height ratio of DMED-HFA to d_4-DMED-HFA.

Table S1. Characteristics of study participants

No.	Gender	Age	Diagnosis	No.	Gender	Age	Diagnosis
1	Female	46	Breast cancer	13	Female	40	Breast cancer
2	Female	46	Breast cancer	14	Female	60	Breast cancer
3	Female	41	Breast cancer	15	Female	56	Breast cancer
4	Female	50	Breast cancer	16	Female	45	Breast cancer
5	Female	49	Breast cancer	17	Female	66	Breast cancer
6	Female	63	Breast cancer	18	Female	51	Breast cancer
7	Female	53	Breast cancer	19	Female	58	Breast cancer
8	Female	49	Breast cancer	20	Female	48	Breast cancer
9	Female	57	Breast cancer	21	Female	60	Breast cancer
10	Female	48	Breast cancer	22	Female	52	Breast cancer
11	Female	48	Breast cancer	23	Female	45	Breast cancer
12	Female	51	Breast cancer	24	Female	40	Breast cancer

Table S2. The abbreviations of lipid compounds.

NO.	FAHFA		FA		HFA	
	**Abbreviation**	**Full name**	**Abbreviation**	**Full name**	**Abbreviation**	**Full name**
**1**	PDAHSA	Pentadecanoic acid-hydroxy stearic acid	PDA	Pentadecanoic acid	HSA	Hydroxy stearic acid
**2**	PAHSA	Palmitic acid-hydroxy stearic acid	PA	Palmitic acid	HPA	Hydroxy palmitic acid
**3**	OAHSA	Oleic acid-hydroxy stearic acid	OA	Oleic acid	HMA	Hydroxy myristic acid
**4**	SAHSA	Stearic acid-hydroxy stearic acid	SA	Stearic acid		
**5**	POHSA	Palmitoleic acid-hydroxy stearic acid	PO	Palmitoleic acid		
**6**	SAHPA	Stearic acid-hydroxy palmitic acid				
**7**	PDAHPA	Pentadecanoic acid-hydroxy palmitic acid				
**8**	PAHPA	Palmitic acid-hydroxy palmitic acid				
**9**	SAHMA	Stearic acid-hydroxy myristic acid				

Table S3. List of FAHFA standards

Family	isomer	Molecular formula	Molecular weight	CAS
PAHSA	13-PAHSA	C34H66O4	538.4961	
	12-PAHSA	C34H66O4	538.4961	1997286-65-3
	10-PAHSA	C34H66O4	538.4961	1636134-73-0
	9-PAHSA	C34H66O4	538.4961	1481636-31-0
	5-PAHSA	C34H66O4	538.4961	1481636-41-2
POHSA	13-POHSA	C34H64O4	536.4805	
	12-POHSA	C34H64O4	536.4805	
	10-POHSA	C34H64O4	536.4805	
	9-POHSA	C34H64O4	536.4805	1481636-43-4
	5-POHSA	C34H64O4	536.4805	2161370-68-7
SAHSA	13-SAHSA	C36H70O4	566.5274	
	12-SAHSA	C36H70O4	566.5274	51350-61-9
	10-SAHSA	C36H70O4	566.5274	
	9-SASHSA	C36H70O4	566.5274	1895916-79-6
	5-SAHSA	C36H70O4	566.5274	
OAHSA	13-OAHSA	C36H68O4	564.5118	
	12-OAHSA	C36H68O4	564.5118	101901-73-9
	10-OAHSA	C36H68O4	564.5118	
	9-OAHSA	C36H68O4	564.5118	154086-90-5
	5-OAHSA	C36H68O4	564.5118	
HDAHSA	10-HDAHSA	C35H68O4	552.5118	
	9-HDAHSA	C35H68O4	552.5118	
	7-HDAHSA	C35H68O4	552.5118	
PDAHSA	10-PDAHSA	C33H64O4	524.4805	
	9-PDAHSA	C33H64O4	524.4805	
	7-PDAHSA	C33H64O4	524.4805	
PDAHPA	10-PDAHPA	C31H60O4	496.4492	
	9-PDAHPA	C31H60O4	496.4492	
	5-PDAHPA	C31H60O4	496.4492	
MAHMA	10-MAHMA	C28H54O4	454.4022	
	9-MAHMA	C28H54O4	454.4022	
	5-MAHMA	C28H54O4	454.4022	
SAHPA	10-SAHPA	C34H66O4	538.4961	
	9-SAHPA	C34H66O4	538.4961	
	5-SAHPA	C34H66O4	538.4961	

MAHPA	10-MAHPA	C30H58O4	482.4335	
	9-MAHPA	C30H58O4	482.4335	
	5-MAHPA	C30H58O4	482.4335	
PAHPA	10-PAHPA	C32H62O4	510.4648	
	9-PAHPA	C32H62O4	510.4648	1636134-70-7
	5-PAHPA	C32H62O4	510.4648	
	3-PAHPA	C32H62O4	510.4648	
PAHMA	10-PAHMA	C30H58O4	482.4335	
	9-PAHMA	C30H58O4	482.4335	
	5-PAHMA	C30H58O4	482.4335	
SAHMA	10-SAHMA	C32H62O4	510.4648	
	9-SAHMA	C32H62O4	510.4648	
	5-SAHMA	C32H62O4	510.4648	

Table S4 The MRM parameters for FAHFA candidates by LC-ESI-MS/MS.

Event	Scan mode	Precursor ion (m/z)	Product ion (m/z)	Collision energy	Event	Scan mode	Precursor ion (m/z)	Product ion (m/z)	Collision energy
1	(+)	595.8	308.5	-37	65	(+)	593.8	322.5	-40
2	(+)	599.8	308.5	-38	66	(+)	597.8	323.5	-37
3	(+)	593.8	308.5	-39	67	(+)	621.8	324.5	-38
4	(+)	597.8	308.5	-40	68	(+)	625.8	325.5	-38
5	(+)	623.8	308.5	-38	69	(+)	637.8	326.5	-38
6	(+)	627.8	308.5	-38	70	(+)	641.8	327.5	-38
7	(+)	609.8	308.5	-38	71	(+)	645.8	328.5	-38
8	(+)	613.8	308.5	-38	72	(+)	649.8	329.5	-38
9	(+)	637.8	308.5	-38	73	(+)	521.5	250.2	-39
10	(+)	641.8	308.5	-38	74	(+)	525.5	250.2	-40
11	(+)	635.8	308.5	-38	75	(+)	565.5	250.2	-38
12	(+)	639.8	308.5	-38	76	(+)	569.5	250.2	-38
13	(+)	633.8	308.5	-38	77	(+)	575.5	250.2	-38
14	(+)	637.8	308.5	-38	78	(+)	579.5	250.2	-38
15	(+)	681.8	308.5	-35	79	(+)	573.5	250.2	-38
16	(+)	685.8	308.5	-35	80	(+)	577.5	250.2	-38
17	(+)	567.6	280.3	-37	81	(+)	623.5	250.2	-38
18	(+)	571.6	280.3	-38	82	(+)	627.5	250.2	-38
19	(+)	565.6	280.3	-39	83	(+)	573.5	248.2	-38
20	(+)	569.6	280.3	-40	84	(+)	577.5	248.2	-38
21	(+)	581.6	280.3	-38	85	(+)	629.5	248.2	-35
22	(+)	585.6	280.3	-38	86	(+)	633.5	248.2	-35
23	(+)	595.6	280.3	-38	87	(+)	621.5	248.2	-35
24	(+)	599.6	280.3	-38	88	(+)	625.5	248.2	-35
25	(+)	609.6	280.3	-38	89	(+)	559.5	246.2	-35
26	(+)	613.6	280.3	-38	90	(+)	563.5	246.2	-35
27	(+)	607.6	280.3	-38	91	(+)	597.5	246.2	-35
28	(+)	611.6	280.3	-38	92	(+)	601.5	246.2	-35
29	(+)	605.6	280.3	-38	93	(+)	551.5	278.2	-38
30	(+)	609.6	280.3	-38	94	(+)	555.5	278.2	-38
31	(+)	631.6	280.3	-38	95	(+)	549.5	278.2	-39
32	(+)	635.6	280.3	-38	96	(+)	553.5	278.2	-40
33	(+)	653.6	280.3	-38	97	(+)	565.5	278.2	-37
34	(+)	657.6	280.3	-38	98	(+)	569.5	278.2	-38
35	(+)	525.5	252.2	-38	99	(+)	579.5	278.2	-38
36	(+)	529.5	252.2	-38	100	(+)	583.5	278.2	-38
37	(+)	553.5	252.2	-38	101	(+)	593.5	278.2	-38

38	(+)	557.5	252.2	-38	102	(+)	597.5	278.2	-38
39	(+)	567.5	252.2	-38	103	(+)	603.5	278.2	-38
40	(+)	571.5	252.2	-38	104	(+)	607.5	278.2	-38
41	(+)	581.5	252.2	-38	105	(+)	601.5	278.2	-38
42	(+)	585.5	252.2	-38	106	(+)	605.5	278.2	-38
43	(+)	625.5	252.2	-38	107	(+)	593.5	306.2	-37
44	(+)	629.5	252.2	-38	108	(+)	597.5	306.2	-38
45	(+)	523.5	224.2	-37	109	(+)	591.5	306.2	-39
46	(+)	527.5	225.2	-38	110	(+)	595.5	306.2	-40
47	(+)	525.5	226.2	-38	111	(+)	607.5	306.2	-38
48	(+)	529.5	227.2	-38	112	(+)	611.5	306.2	-38
49	(+)	539.5	228.2	-38	113	(+)	631.5	306.2	-38
50	(+)	543.5	229.2	-38	114	(+)	635.5	306.2	-38
51	(+)	553.5	230.2	-38	115	(+)	687.5	306.2	-38
52	(+)	557.5	231.2	-38	116	(+)	691.5	306.2	-38
53	(+)	551.5	232.2	-38	117	(+)	679.5	306.2	-38
54	(+)	555.5	233.2	-38	118	(+)	683.5	306.2	-38
55	(+)	597.5	234.2	-38	119	(+)	605.5	304.2	-38
56	(+)	601.5	235.2	-38	120	(+)	609.5	304.2	-38
57	(+)	581.6	294.3	-37	121	(+)	631.5	304.2	-38
58	(+)	585.6	295.3	-38	122	(+)	635.5	304.2	-38
59	(+)	579.6	296.3	-39	123	(+)	629.5	304.2	-38
60	(+)	583.6	297.3	-40	124	(+)	633.5	304.2	-38
61	(+)	619.6	298.3	-38	125	(+)	677.5	304.2	-38
62	(+)	623.6	299.3	-35	126	(+)	681.5	304.2	-38
63	(+)	667.6	300.3	-35	127	(+)	627.5	302.2	-38
64	(+)	671.6	301.3	-39	128	(+)	631.5	302.2	-38

Table S5. Detected FAHFAs in tissues.

No.	Analytes	Precursor ion(m/z)	Product ion(m/z)[1]	Retention time(min)	Formula(unlabeled)	Molecular weight (unlabeled)
1	9-PDAHSA	595.5	**308.3**/353.4	14.468	C33H64O4	524.4805
2	7-PDAHSA	595.5	**308.3**/353.6	15.590	C33H64O4	524.4805
3	15-PDAHSA	595.5	**308.3**/353.8	11.943	C33H64O4	524.4805
4	14-PDAHSA	595.5	**308.3**/353.9	12.242	C33H64O4	524.4805
5	13-PDAHSA	595.5	**308.3**/353.10	12.520	C33H64O4	524.4805
6	12-PDAHSA	595.5	**308.3**/353.11	13.497	C33H64O4	524.4805
7	11-PDAHSA	595.5	**308.3**/353.12	13.725	C33H64O4	524.4805
8	10-PDAHSA	595.5	**308.3**/353.13	13.999	C33H64O4	524.4805
9	8-PDAHSA	595.5	**308.3**/353.14	15.014	C33H64O4	524.4805
10	6-PDAHSA	595.5	**308.3**/353.15	16.049	C33H64O4	524.4805
11	13-PAHSA	609.6	**308.3**/353.16	14.629	C34H66O4	538.4961
12	12-PAHSA	609.6	**308.3**/353.18	14.977	C34H66O4	538.4961
13	10-PAHSA	609.6	**308.3**/353.20	15.960	C34H66O4	538.4961
14	9-PAHSA	609.6	**308.3**/353.22	16.512	C34H66O4	538.4961
15	5-PAHSA	609.6	**308.3**/353.24	18.808	C34H66O4	538.4961
16	3-PAHSA	609.6	**308.3**/353.26	19.957	C34H66O4	538.4961
17	15-PAHSA	609.6	**308.3**/353.28	13.927	C34H66O4	538.4961
18	14-PAHSA	609.6	**308.3**/353.29	14.156	C34H66O4	538.4961
19	11-PAHSA	609.6	**308.3**/353.30	15.464	C34H66O4	538.4961
20	8-PAHSA	609.6	**308.3**/353.31	17.081	C34H66O4	538.4961

21	7-PAHSA	609.6	**308.3**/353.32	17.628	C34H66O4	538.4961
22	6-PAHSA	609.6	**308.3**/353.33	18.187	C34H66O4	538.4961
23	13-OAHSA	635.6	**308.3**/353.34	14.858	C36H68O4	564.5118
24	12-OAHSA	635.6	**308.3**/353.36	15.275	C36H68O4	564.5118
25	10-OAHSA	635.6	**308.3**/353.38	16.269	C36H68O4	564.5118
26	9-OAHSA	635.6	**308.3**/353.40	16.788	C36H68O4	564.5118
27	11-OAHSA	635.6	**308.3**/353.42	15.737	C36H68O4	564.5118
28	12-SAHSA	637.6	**308.3**/353.45	19.351	C36H70O4	566.5274
29	10-SAHSA	637.6	**308.3**/353.47	20.464	C36H70O4	566.5274
30	9-SAHSA	637.6	**308.3**/353.49	20.994	C36H70O4	566.5274
31	5-SAHSA	637.6	**308.3**/353.51	23.368	C36H70O4	566.5274
32	15-SAHSA	637.6	**308.3**/353.53	18.336	C36H70O4	566.5274
33	14-SAHSA	637.6	**308.3**/353.54	18.526	C36H70O4	566.5274
34	11-SAHSA	637.6	**308.3**/353.55	19.860	C36H70O4	566.5274
35	8-SAHSA	637.6	**308.3**/353.56	21.560	C36H70O4	566.5274
36	7-SAHSA	637.6	**308.3**/353.57	22.221	C36H70O4	566.5274
37	6-SAHSA	637.6	**308.3**/353.58	22.733	C36H70O4	566.5274
38	13-POHSA	607.6	**308.3**/353.59	11.775	C34H64O4	536.4805
39	12-POHSA	607.6	**308.3**/353.61	11.775	C34H64O4	536.4805
40	10-POHSA	607.6	**308.3**/353.63	10.921	C34H64O4	536.4805
41	9-POHSA	607.6	**308.3**/353.65	11.455	C34H64O4	536.4805
42	10-SAHPA	609.6	**235.2**/280.2	16.522	C34H66O4	538.4961
43	9-SAHPA	609.6	**235.2**/280.2	16.983	C34H66O4	538.4961
44	5-SAHPA	609.6	**235.2**/280.2	19.235	C34H66O4	538.4961

45	13-SAHPA	609.6	**235.2**/280.2	14.800	C34H66O4	538.4961
46	12-SAHPA	609.6	**235.2**/280.2	15.482	C34H66O4	538.4961
47	11-SAHPA	609.6	**235.2**/280.2	16.013	C34H66O4	538.4961
48	8-SAHPA	609.6	**235.2**/280.2	17.525	C34H66O4	538.4961
49	7-SAHPA	609.6	**235.2**/280.2	17.985	C34H66O4	538.4961
50	6-SAHPA	609.6	**235.2**/280.2	18.636	C34H66O4	538.4961
51	10-PDAHPA	567.5	**235.2**/280.2	10.295	C31H60O4	496.4492
52	9-PDAHPA	567.5	**235.2**/280.2	10.792	C31H60O4	496.4492
53	5-PDAHPA	567.5	**235.2**/280.2	12.908	C31H60O4	496.4492
54	13-PDAHPA	567.5	**235.2**/280.2	8.969	C31H60O4	496.4492
55	12-PDAHPA	567.5	**235.2**/280.2	9.440	C31H60O4	496.4492
56	11-PDAHPA	567.5	**235.2**/280.2	9.979	C31H60O4	496.4492
57	8-PDAHPA	567.5	**235.2**/280.2	11.270	C31H60O4	496.4492
58	7-PDAHPA	567.5	**235.2**/280.2	11.727	C31H60O4	496.4492
59	6-PDAHPA	567.5	**235.2**/280.2	12.253	C31H60O4	496.4492
60	10-PAHPA	581.5	**235.2**/280.2	12.324	C32H62O4	510.4648
61	9-PAHPA	581.5	**235.2**/280.2	12.785	C32H62O4	510.4648
62	5-PAHPA	581.5	**235.2**/280.2	15.029	C32H62O4	510.4648
63	3-PAHPA	581.5	**235.2**/280.2	16.276	C32H62O4	510.4648
64	13-PAHPA	581.5	**235.2**/280.2	10.849	C32H62O4	510.4648
65	12-PAHPA	581.5	**235.2**/280.2	11.237	C32H62O4	510.4648
66	11-PAHPA	581.5	**235.2**/280.2	11.439	C32H62O4	510.4648
67	8-PAHPA	581.5	**235.2**/280.2	13.279	C32H62O4	510.4648
68	7-PAHPA	581.5	**235.2**/280.2	13.845	C32H62O4	510.4648

69	6-PAHPA	581.5	**235.2**/280.2	14.376	C32H62O4	510.4648
70	10-SAHMA	581.6	**207.3**/252.3	13.252	C32H62O4	510.4648
71	9-SAHMA	581.6	**207.3**/252.3	14.026	C32H62O4	510.4648
72	5-SAHMA	581.6	**207.3**/252.3	7.898	C32H62O4	510.4648

[1] Highlighted ions are predominant product ions

Table S6. Structures of FAHFAs detected in breast tissues.

NO.	Name	Formula	Structure
1	PDAHSA	C33H64O4	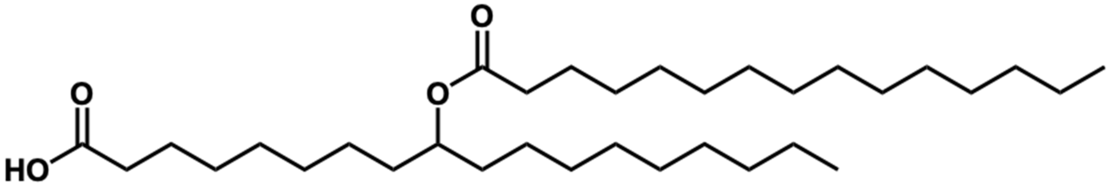
2	PAHSA	C34H66O4	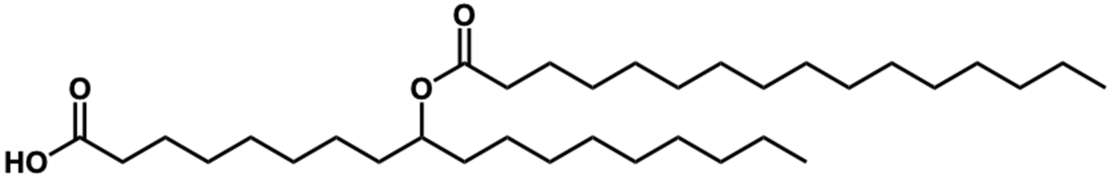
3	OAHSA	C36H68O4	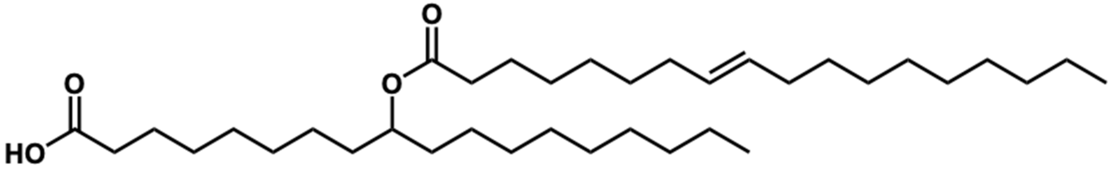
4	SAHSA	C36H70O4	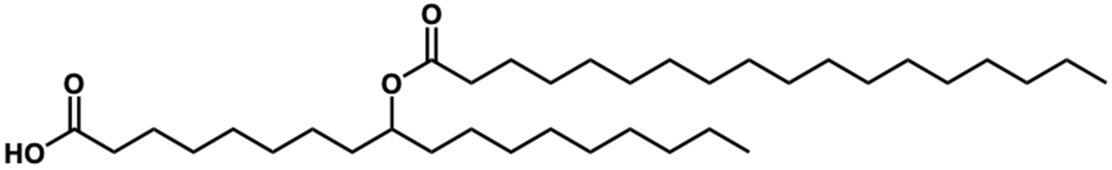
5	POHSA	C34H64O4	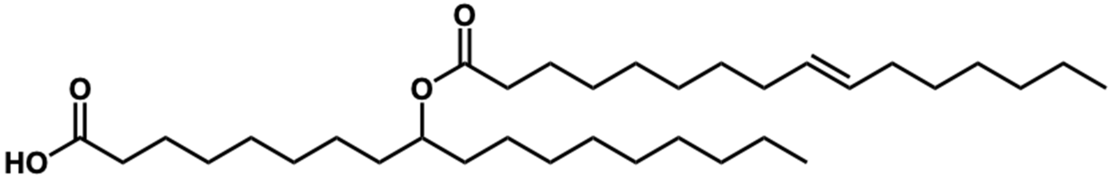

6	SAHPA	C34H66O4	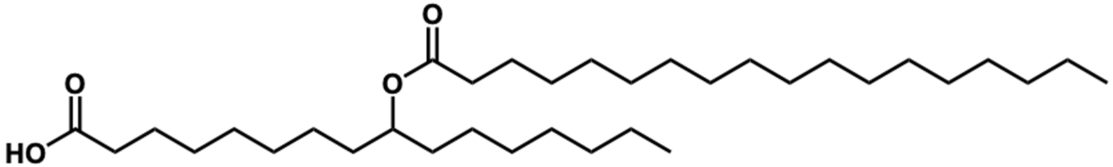
7	PDAHPA	C31H60O4	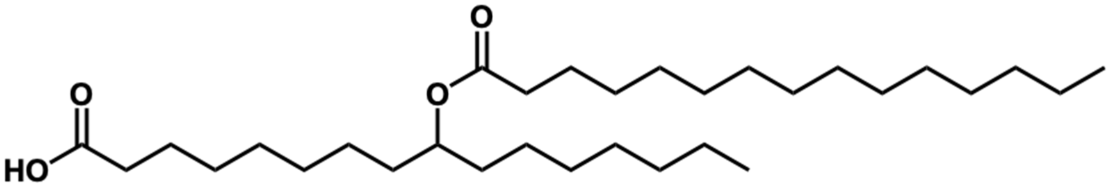
8	PAHPA	C32H62O4	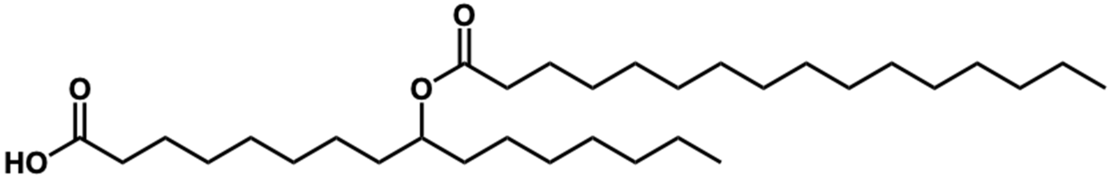
9	SAHMA	C32H62O4	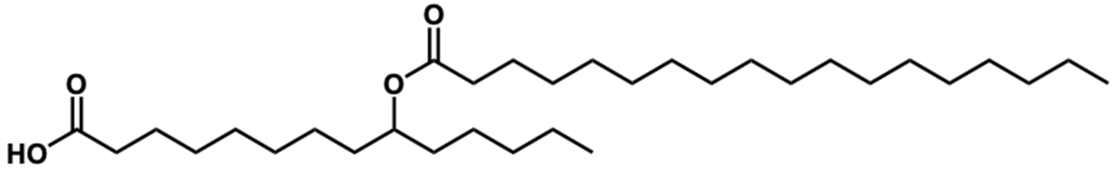

Table S7. Measured levels of FAHFA isomers were detected in breast tissues, including absolute quantitative and semi-quantitative analysis in tumor and adjacent normal tissues. The data represent the mean and standard deviation (SD) of the measured levels of FAHFAs.

Family	isomer	Tumor tissue (ng/g)	Adjacent normal tissue (ng/g)
PDAHSA	9-PDAHSA	0.12 ± 0.02	0.14 ± 0.08
	7-PDAHSA	0.11 ± 0.02	0.11 ± 0.03
PAHSA	13-PAHSA	0.35 ± 0.07	0.45 ± 0.23
	12-PAHSA	0.32 ± 0.05	0.40 ± 0.20
	10-PAHSA	1.24 ± 0.37	1.54 ± 0.12
	9-PAHSA	1.16 ± 0.49	0.99 ± 0.65
	5-PAHSA	0.11 ± 0.01	0.15 ± 0.09
	3-PAHSA	0.42 ± 0.21	0.43 ± 0.25
OAHSA	13-OAHSA	0.23 ± 0.16	0.10 ± 0.07
	12-OAHSA	0.15 ± 0.11	0.13 ± 0.08
	10-OAHSA	0.49 ± 0.38	0.29 ± 0.19
	9-OAHSA	1.91 ± 0.16	0.42 ± 0.02
SAHSA	12-SAHSA	0.30 ± 0.07	0.33 ± 0.13
	10-SAHSA	0.54 ± 0.14	0.88 ± 0.52
	9-SAHSA	0.15 ± 0.02	0.16 ± 0.07
	5-SAHSA	0.10 ± 0.03	0.09 ± 0.06
POHSA	13-POHSA	0.11 ± 0.02	0.11 ± 0.02
	12-POHSA	0.08 ± 0.02	0.08 ± 0.02
	10-POHSA	0.14 ± 0.05	0.13 ± 0.10
	9-POHSA	0.38 ± 0.02	0.16 ± 0.06
SAHPA	10-SAHPA	0.36 ± 0.07	0.44 ± 0.23
	9-SAHPA	0.40 ± 0.12	0.40 ± 0.23
	5-SAHPA	0.33 ± 0.11	0.33 ± 0.16
PDAHPA	10-PDAHPA	0.14 ± 0.02	0.15 ± 0.04
	9-PDAHPA	0.13 ± 0.02	0.15 ± 0.05
	5-PDAHPA	0.11 ± 0.02	0.12 ± 0.03
PAHPA	10-PAHPA	0.24 ± 0.04	0.27 ± 0.11
	9-PAHPA	0.23 ± 0.06	0.19 ± 0.05
	5-PAHPA	0.16 ± 0.04	0.18 ± 0.08
	3-PAHPA	0.36 ± 0.02	0.46 ± 0.22
SAHMA	10-SAHMA	0.19 ± 0.04	0.21 ± 0.15
	9-SAHMA	0.11 ± 0.03	0.13 ± 0.11
	5-SAHMA	0.25 ±0.02	0.09 ± 0.04
**Semi-quantitative**			

PDAHSA	15-PDAHSA	0.16 ± 0.14	0.17 ± 0.11
	14-PDAHSA	0.16 ± 0.12	0.21 ± 0.20
	13-PDAHSA	0.10 ± 0.07	0.13 ± 0.09
	12-PDAHSA	0.08 ± 0.04	0.12 ± 0.08
	11-PDAHSA	0.09 ± 0.03	0.12 ± 0.09
	10-PDAHSA	0.10 ± 0.03	0.15 ± 0.11
	8-PDAHSA	0.10 ± 0.04	0.12 ± 0.09
	6-PDAHSA	0.10 ± 0.09	0.09 ± 0.06
PAHSA	15-PAHSA	1.55 ± 1.15	1.38 ± 0.95
	14-PAHSA	1.64 ± 0.93	1.52 ± 0.94
	11-PAHSA	2.58 ± 1.45	2.26 ± 1.45
	8-PAHSA	1.17 ± 0.47	1.34 ± 0.96
	7-PAHSA	0.84 ± 0.62	0.78 ± 0.47
	6-PAHSA	1.31 ± 1.23	0.72 ± 0.53
OAHSA	11-OAHSA	2.30 ± 1.95	0.72 ± 0.43
SAHSA	15-SAHSA	0.46 ± 0.46	0.43 ± 0.34
	14-SAHSA	0.46 ± 0.35	0.56 ± 0.46
	11-SAHSA	0.41 ± 0.12	0.64 ± 0.48
	8-SAHSA	0.32 ± 0.13	0.35 ± 0.24
	7-SAHSA	0.26 ± 0.25	0.25 ± 0.22
	6-SAHSA	0.24 ± 0.15	0.16 ± 0.15
SAHPA	13-SAHPA	3.84 ± 2.97	3.37 ± 2.26
	12-SAHPA	2.19 ± 1.85	2.68 ± 1.71
	11-SAHPA	1.59 ± 0.52	2.15 ± 1.29
	8-SAHPA	1.73 ± 0.51	2.32 ± 1.41
	7-SAHPA	2.80 ± 1.09	2.56 ± 1.62
	6-SAHPA	1.70 ± 0.51	1.71 ± 1.07
PDAHPA	13-PDAHPA	0.42 ± 0.21	0.73 ± 0.42
	12-PDAHPA	0.26 ± 0.14	0.51 ± 0.27
	11-PDAHPA	0.26 ± 0.14	0.44 ± 0.25
	8-PDAHPA	0.25 ± 0.13	0.46 ± 0.26
	7-PDAHPA	0.22 ± 0.10	0.41 ± 0.25
	6-PDAHPA	0.18 ± 0.10	0.31 ± 0.20
PAHPA	13-PAHPA	3.48 ± 2.83	2.84 ± 2.18
	12-PAHPA	1.31 ± 0.61	1.42 ± 0.84
	11-PAHPA	1.02 ± 0.30	1.27 ± 0.80
	8-PAHPA	1.13 ± 0.42	1.21 ± 0.68
	7-PAHPA	2.68 ± 1.27	1.75 ± 0.99
	6-PAHPA	0.85 ± 0.26	1.05 ± 0.64

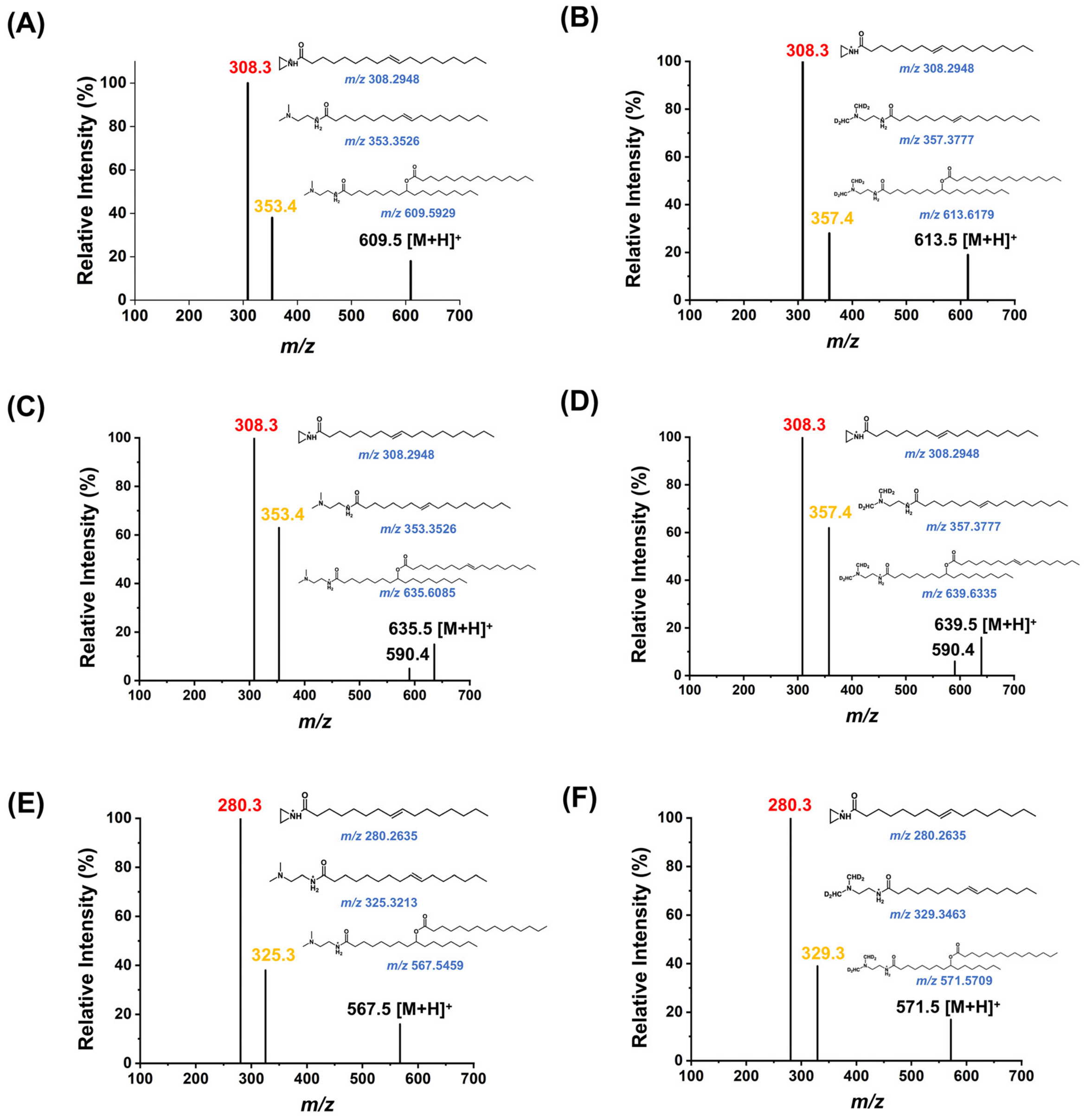

Figure S1. Product ions spectra of DMED/d_4-DMED labeled FAHFAs. (A) DMED labeled 9-PAHSA; (B) d_4-DMED labeled 9-PAHSA; (C) DMED labeled 9-OAHSA; (D) d_4-DMED labeled 9-OAHSA; (E) DMED labeled 9-PDAHPA; (F) d_4-DMED labeled 9-PDAHPA. Highlighted in red are predominant product ions, highlighted in yellow are product ions that aid in qualitative analysis, and highlighted in blue are the theoretical m/z.

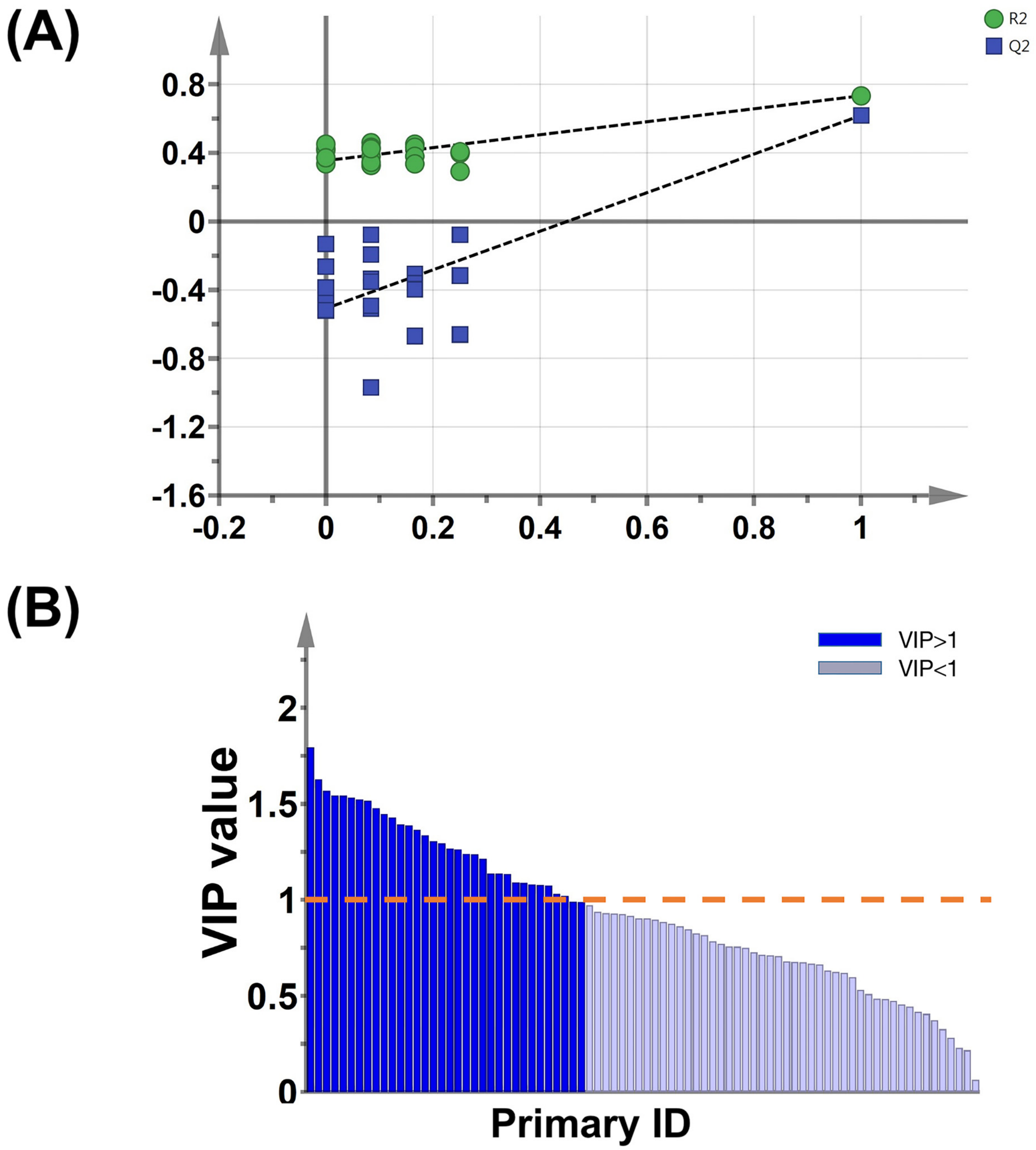

Figure S2. Permutation (A) and VIP distribution (B) of the OPLS-DA module.

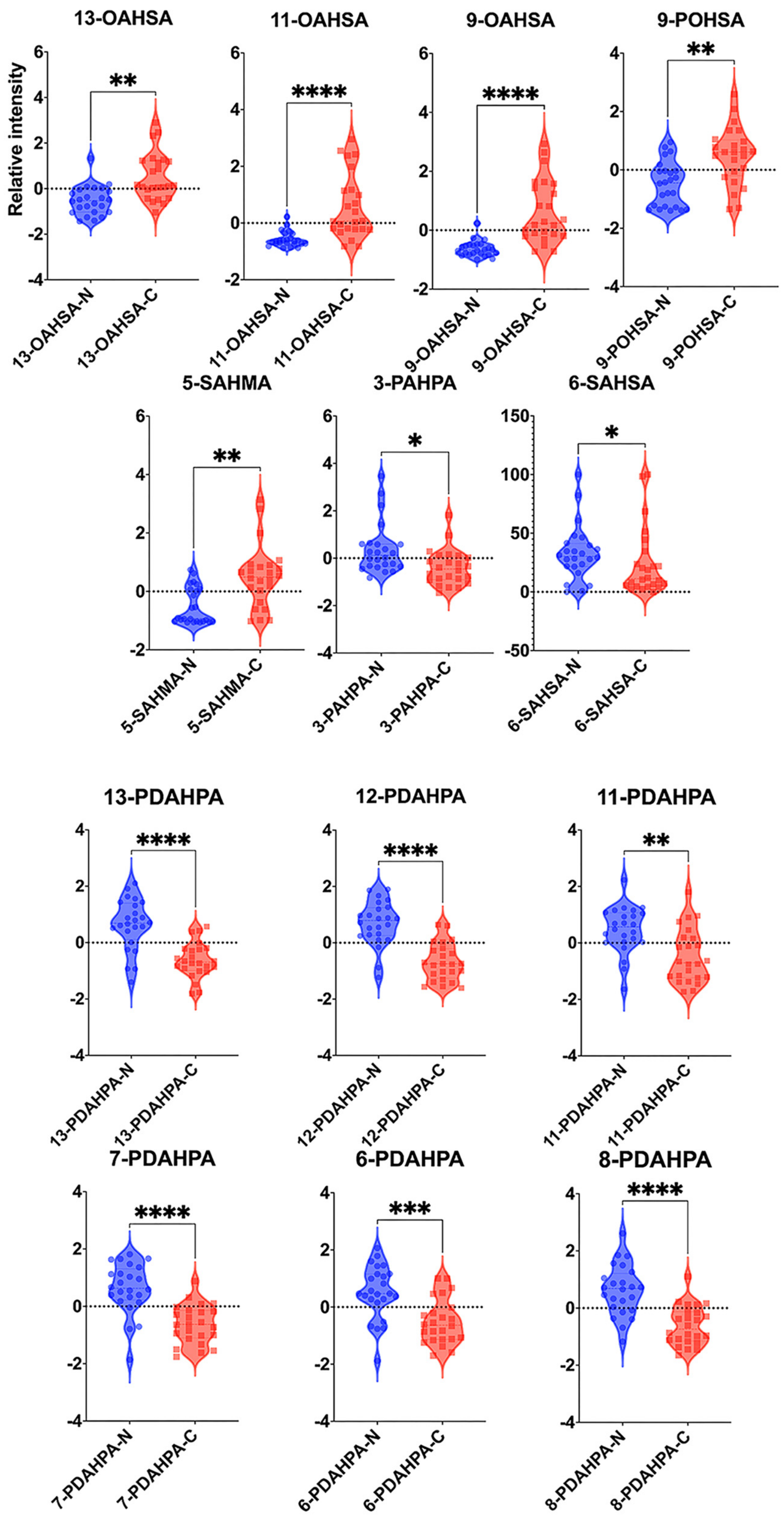

Figure S3. The violin scatter plots of FAHFAs isomers with significant differences in tumor and adjacent normal tissue of cancer. N, normal tissue; C, tumor tissue. $*$, $p < 0.05$; $**$, $p < 0.01$; $***$, $p < 0.001$; $****$, $p < 0.0001$.

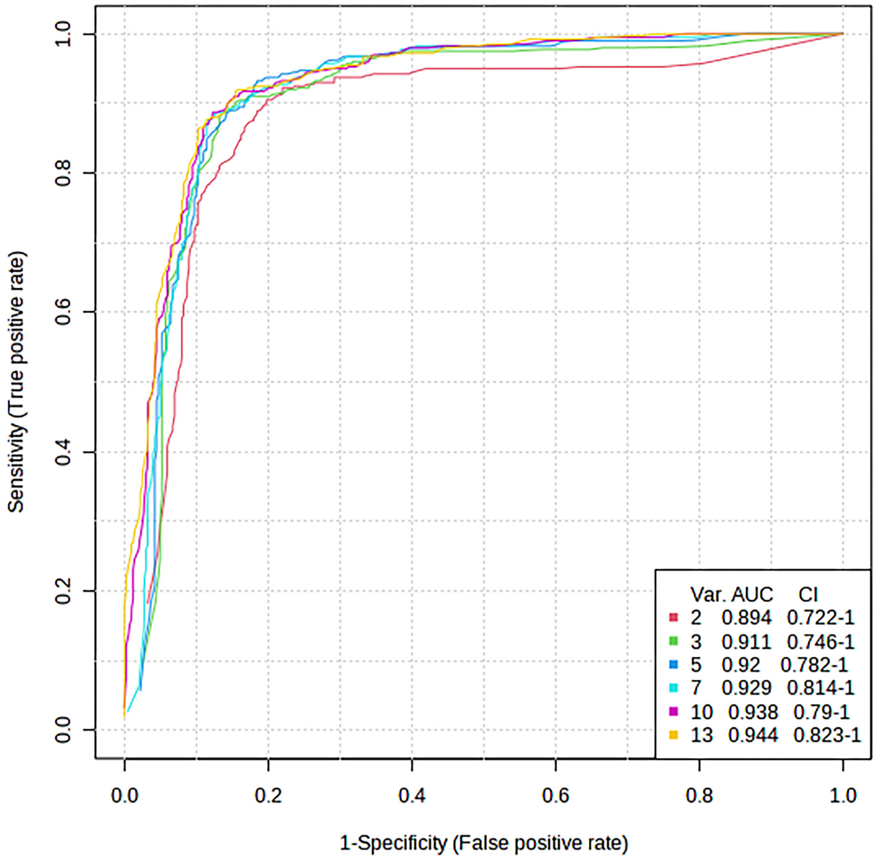

Figure S4. Multivariate ROC model based on differentiated FAHFAs using Random Forest as a construction method. The AUC in the plots corresponds to ROC models with different combinations of differentiated FAHFAs.

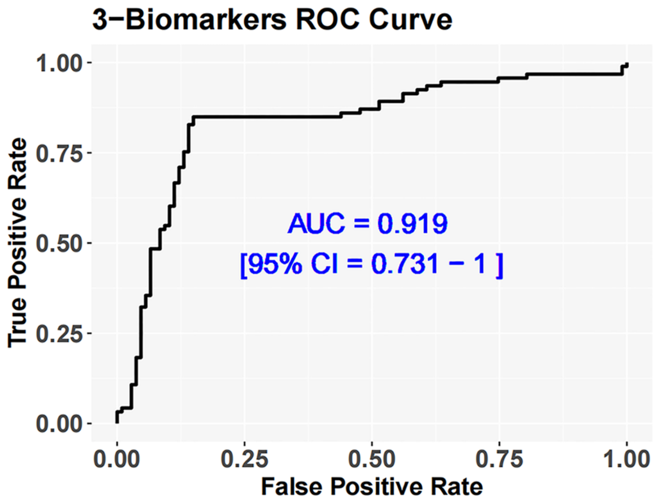

Figure S5. Multivariate ROC analysis based on 9-OAHSA, 11-OAHSA, and 12-PDAHPA.
